# Lgt Processing Is an Essential Step in *Streptococcus suis* Lipoprotein Mediated Innate Immune Activation

**DOI:** 10.1371/journal.pone.0022299

**Published:** 2011-07-19

**Authors:** Paul J. Wichgers Schreur, Johanna M. J. Rebel, Mari A. Smits, Jos P. M. van Putten, Hilde E. Smith

**Affiliations:** 1 Central Veterinary Institute, Wageningen UR, Lelystad, The Netherlands; 2 Wageningen Livestock Research, Wageningen UR, Lelystad, The Netherlands; 3 Department of Infectious Diseases and Immunology, Utrecht University, Utrecht, The Netherlands; Cairo University, Egypt

## Abstract

**Background:**

*Streptococcus suis* causes invasive infections in pigs and occasionally in humans. The host innate immune system plays a major role in counteracting *S. suis* infections. The main components of *S. suis* able to activate the innate immune system likely include cell wall constituents that may be released during growth or after cell wall integrity loss, however characterization of these components is still limited.

**Methology/Principal Findings:**

A concentrated very potent innate immunity activating supernatant of penicillin-treated *S. suis* was SDS-PAGE fractionated and tested for porcine peripheral blood mononucleated cell (PBMC) stimulating activity using cytokine gene transcript analysis. More than half of the 24 tested fractions increased IL-1β and IL-8 cytokine gene transcript levels in porcine PBMCs. Mass spectrometry of the active fractions indicated 24 proteins including 9 lipoproteins. Genetic inactivation of a putative prolipoprotein diacylglyceryl transferase (Lgt) gene resulted in deficient lipoprotein synthesis as evidenced by palmitate labeling. The Lgt mutant showed strongly reduced activation of porcine PBMCs, indicating that lipoproteins are dominant porcine PBMC activating molecules of *S. suis*.

**Conclusion/Significance:**

This study for the first time identifies and characterizes lipoproteins of *S. suis* as major activators of the innate immune system of the pig. In addition, we provide evidence that Lgt processing of lipoproteins is required for lipoprotein mediated innate immune activation.

## Introduction

*Streptococcus suis* causes severe infections in pigs, including meningitis, septicemia, endocarditis, pneumonia and arthritis. Occasionally, *S. suis* infects humans as well, resulting in comparable disease manifestations as are seen in pigs [1], [2], [3]. To date, 33 serotypes of *S. suis* have been described based on differences in polysaccharide capsule. Isolates even of the same serotype may vary in virulence. The majority of isolates that causes disease belong to serotype 2, although in Europe serotype 9 isolates are emerging [4], [5], [6].

Based on the existence of a strong inflammatory response during an acute *S. suis* infection, a significant activation of innate immunity is expected early after infection. The innate immune system uses pattern recognition receptors (PRRs) to recognize pathogen associated molecular patterns (PAMPs) of microbes. One group of PRRs able to sense a diverse set of bacterial PAMPs is the Toll-like receptor (TLR) family. Activation of these TLRs results in nuclear translocation of transcription factors (e.g. nuclear factor kappa B, NF-κB) which ultimately causes enhanced production of pro-inflammatory cytokines, chemokines and antimicrobial peptides. Besides these direct mechanisms to eliminate invading microbes, the innate immune system plays a decisive role in initiating and strengthening humoral and cell-mediated protection.

The capsule of *S. suis* may be one of the first structures to be recognized by the innate immune system. However, capsule by itself is a poor activator of the innate immune system [7]. Capsule-deficient *S. suis* strains display even higher levels of innate activation compared to wild type strains in human monocytes and macrophages [7], [8]. The main components of *S. suis* involved in activating the innate immune system therefore likely include cell wall or cell membrane constituents. Indeed, cell wall extracts of *S. suis* have been shown to be potent cytokine inducers in murine macrophages, human endothelial brain cells, human monocytes and in a porcine whole blood model [8], [9], [10], [11]. Furthermore, we recently provided evidence that components of *S. suis* released after cell wall integrity loss specifically activate the human TLR2/6 complex that mostly recognizes bacterial lipoproteins [12].

Lipoproteins of Gram-positive bacteria are processed by two key enzymes; the prolipoprotein diacylglyceryl transferase (Lgt) enzyme and the lipoprotein signal peptidase (Lsp) enzyme. The Lgt enzyme recognizes a so-called lipobox motif (LXXC) in the C-terminal region of the signal peptide of a premature lipoprotein and transfers a diacylglyceryl moiety to the cysteine residue of the lipobox [13], [14]. Subsequently, the Lsp enzyme cleaves the signal peptide resulting in a mature lipoprotein [15], [16]. Lipid modification of Gram-positive bacterial lipoproteins via Lgt has been described to be essential for innate immune activation [17], [18].

The objective of this study was to identify components of *S. suis* that activate porcine peripheral blood mononucleated cells (PBMCs). We used mass spectrometry and genetically defined lipoprotein-processing defective strains as research instruments.

## Results

### *S. suis* activates porcine PBMCs efficiently

**Figure 1:**
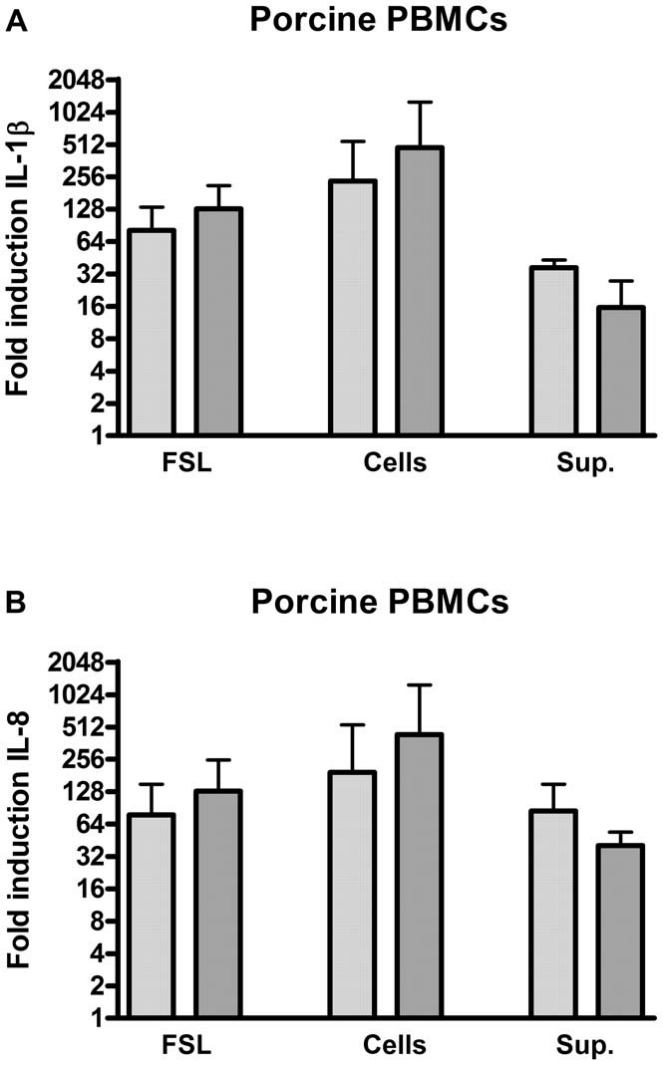
Porcine PBMC stimulation with wild type *S. suis*. Porcine PBMCs were stimulated with whole *S. suis* strain 8067 bacteria (cells) in the presence of penicillin or with supernatant (sup.) derived from penicillin treated bacteria. At 2 (light grey bar) and 4 h (dark grey bar) post stimulation IL-1β (A) and IL-8 (B) mRNA expression levels were determined by quantitative real time PCR. The diacylatedlipopeptide FSL was used as a positive control. Data represent fold inductions calculated by dividing the normalized cytokine levels of stimulated cells by the normalized cytokine levels of medium-stimulated negative control cells. Values represent the mean ± SD of two experiments performed in duplicate.

Porcine PBMCs were isolated from pig blood and incubated with *S. suis* and collected bacterial culture supernatant. Penicillin was used to enhance the possible release of PBMC activating components. PBMC activation was determined by measuring changes in IL-1β and IL-8 mRNA transcripts using qRT-PCR. Stimulation of PBMCs with penicillin treated *S. suis* increased IL-1β and IL-8 cytokine transcripts to similar levels as obtained after stimulation with FSL-1, a synthetic lipopeptide (Fig. 1). Activating components were not exclusively cell bound since bacterial culture supernatant stimulated the PBMCs as well (Fig. 1). These results indicate that penicillin-treated *S. suis* is sensed efficiently by the porcine innate immune system and that activating component(s) are released into the supernatant.

### Identification of innate immunity activating proteins

**Figure 2:**
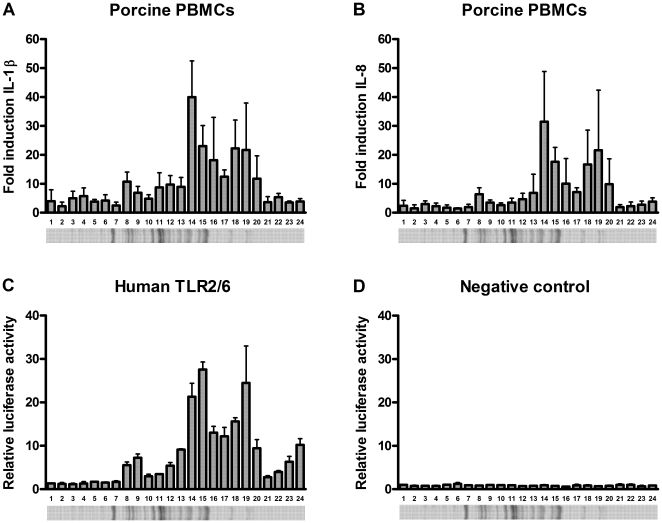
Porcine PBMC and human TLR2/6 stimulation of innate immunity activating fractions. Porcine PBMCs were stimulated with an innate immunity activating fraction of *S. suis* strain 8067 (concentrated supernatant of penicillin treated bacteria) subdivided into 24 fractions of different molecular sizes. At 4 h post stimulation IL-1β (A) and IL-8 (B) mRNA expression levels were determined by quantitative real time PCR. HeLa 57A cells expressing human TLR2/6 (C), and control cells transfected with vector without insert (D) were stimulated (5 h) with the same 24 fractions. Data represent relative fold activation calculated by dividing the normalized test samples by the normalized activity of medium-stimulated negative control samples. Values represent the mean ± SD of two experiments performed in duplicate. Fractions that induced >5 fold porcine PBMC activation were analyzed by mass spectrometry (Table 1).

To gain more insights into the nature of the porcine PBMC activating component(s), we concentrated the supernatant of penicillin-treated *S. suis* and size fractionated it into 24 fractions by SDS-PAGE. The obtained fractions were analyzed for their ability to stimulate porcine PBMCs. More than half of the fractions increased IL-1β and IL-8 cytokine transcript levels as measured by qRT-PCR (Fig. 2A, B). The kinetics of the changes in IL-1β and IL-8 mRNA were very similar. The fractions that caused a more than 5-fold increase in IL-1β and IL-8 mRNA were individually analyzed by mass spectrometry. Mascot scores were determined using the identified peptides in all the fractions simultaneously to increase the sensitivity and specificity of the analysis. A total of 24 *S. suis* proteins with MASCOT scores >50 (Table 1) were identified. Among these 24 proteins, nine (37.5%) putative lipoproteins were present, including two lipoproteins previously shown to be recognized by porcine convalescent sera [19], [20]. In the genome of *S. suis* strain P1/7, 45 putative lipoprotein coding genes are present (Table S1, [21]) which corresponds to 2.5% of the proteome [21]. This large enrichment of lipoproteins in the porcine PBMC activating fractions suggests that *S. suis* lipoproteins contribute to the observed PBMC activation.

**Table 1:** Identified proteins within porcine PBMC activating fractions using mass spectrometry.

Nr.	Identified protein	NCBI accession number	SSU in P1/7	Coverage (%)	# Peptides matched	# Amino acids	Molecular weight (kD)	Isoelectric point	Mascot score
1	Glyceraldehyde-3-phosphate dehydrogenase	123967422	153	19	30	335	35.6	5.58	1106.37
2 *	Basic membrane lipoprotein	81096738	934	12	11	355	36.3	4.93	498.48
3 *	High-affinity metal binding protein precursor	146319740	1869	8	6	317	35.5	5.38	298.76
4 *	Amino acid ABC transporter, amino acid-binding protein	146318206	503	22	7	280	31.3	4.67	293.96
5	Enoyl-CoA hydratase	146319463	1609	13	5	263	28.6	5.41	271.41
6	Mannose-specific PTS IID	146321637	1585	23	7	303	33.2	8.07	262.69
7 *	Amino acid ABC transporter, periplasmic protein	146318671	875	22	5	266	28.0	4.65	262.00
8	3-oxoacyl-(acyl-carrier-protein) reductase	81097246	1603	26	4	244	25.6	5.47	243.33
9 *	Hypothetical protein SSU98_1558	146321405	1364	7	8	380	40.1	4.96	218.37
10	L-lactate dehydrogenase	81096123	927	8	6	327	35.4	5.24	214.96
11	Triosephosphate isomerase	146318185	483	12	3	250	26.6	4.79	204.04
12	Fructose-bisphosphate aldolase	146320177	312	18	4	293	31.1	4.98	200.87
13	Ribosomal protein L1, bacterial and chloroplast form	81097390	1164	25	5	186	19.8	9.35	172.66
14	Phosphoglycerate kinase	146317815	154	8	2	399	42.0	4.96	161.88
15 *	Parvulin-like peptidyl-prolyl isomerase	146321102	1078	9	2	255	27.9	5.16	146.14
16	Elongation factor Ts	81177336	1770	9	2	346	37.2	4.79	134.24
17	Phosphoglycerate mutase 1	81176996	1451	14	3	230	26.0	5.30	128.50
18 *	ABC-type metal ion transport system, periplasmic component/surface antigen	146321629	1577	12	2	283	31.0	4.67	117.48
19	D-alanine–D-alanine ligase	81096718	1184	6	2	348	38.7	4.79	110.48
20 *	Extracellular solute-binding protein, family 3	81096925	1853	11	2	267	28.6	4.58	96.38
21	Hypothetical protein SSU98_0389	146320236	361	19	2	132	15.1	5.94	95.79
22 *	ABC transporter substrate-binding protein - maltose/maltodextrin	81097038	1915g	11	2	249	26.6	4.97	95.45
23	Glucokinase ROK	81096801	775	8	2	319	33.4	4.97	84.67
24	Hypothetical protein SSU98_0901	146320748	839	11	2	201	23.4	5.05	67.30

Mass spectrometry identified proteins (of strain 8067) within molecular size fractionated innate immunity stimulating fractions containing >5 fold porcine PBMC activating capacity. The peptides were searched against the SWISS-PROT and non-redundant NCBI database to identify the proteins. In total 24 proteins were identified and ranked by MASCOT score, which indicates the reliability of the identification which is partly correlated with the protein quantity. The corresponding annotated proteins in strain P1/7 are shown in lane 4.

*Annotated as lipoprotein.

### Porcine PBMC activating fractions also activate human TLR2/6 expressing HeLa cells

To investigate the specificity of the (lipo)proteins for porcine PBMC activation, we analyzed the same fractions as used in the PBMC experiment to stimulate HeLa cells expressing human TLR2/6 and a NF-κB luciferase reporter [22]. Human TLR2/6 recognizes bacterial lipoproteins including those of *S. suis* [12]. As shown in Fig. 2C, all fractions able to initiate a porcine IL-1β and IL-8 response (Fig. 2A, B) also activated the TLR2/6-expressing HeLa cells (Fig. 2C), while fractions with low activity yielded a poor response in both porcine PBMCs and human TLR2/6-expressing cells. None of the tested fractions was able to activate transfected HeLa cells lacking TLR2/6 expression (Fig. 2D). The comparable activation of the porcine PBMCs and the human TLR2/6 cell system strongly suggests that lipoproteins have a major role in activating porcine PBMCs, although these results do not exclude that also non-lipoproteins activate porcine PBMCs.

### Generation and characterization of a *S. suis*Δ*lgt* mutant

**Figure 3:**
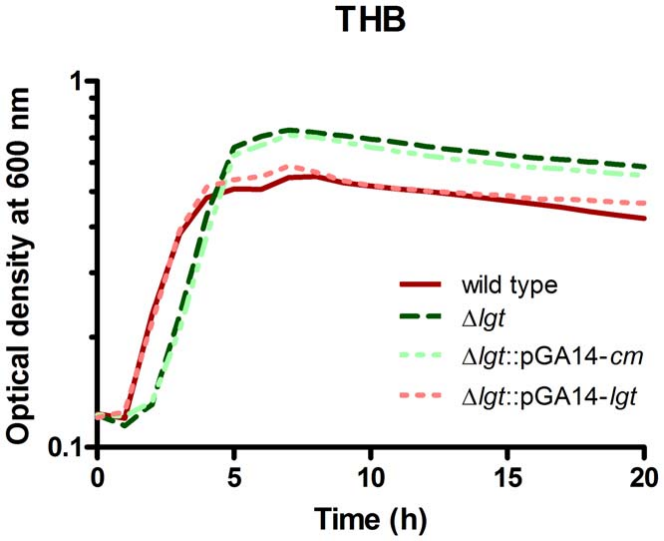
Growth of wild type and Δ*lgt* mutant bacteria. Growth of wild type, Δ*lgt* mutant, and the complemented Δ*lgt* mutant (Δ*lgt*::pGA14-*cm*; Δ*lgt*::pGA14-*lgt*) bacteria was assessed in THB by following optical densities in time.

To distinguish between lipoprotein and non-lipoprotein mediated innate immune activation of porcine PBMCs, we constructed a mutant *S. suis* serotype 9 isolate deficient in the expression of the lipoprotein processing enzyme Lgt. Lgt in Gram-positive bacteria is required for lipid modification of the cysteine residue present within the lipobox of prelipoproteins. In the genome of *S. suis* serotype 2 strain P1/7 gene SSU_1418 had been annotated to encode the Lgt protein. This putative Lgt protein showed 67% amino acid sequence identity to the Lgt protein of *S. pneumoniae* strain D39 [23]. The *lgt* gene is the second gene transcribed of an operon expressing 4 genes also encoding two putative exported proteins and a phosphorylase enzyme. We inactivated the corresponding *lgt* gene in *S. suis* serotype 9 strain 8067 by homologous recombination generating Δ*lgt* mutant bacteria. A positive control was made by re-introducing an intact *lgt* gene in the Δ*lgt* mutant strain by plasmid complementation generating Δ*lgt*::pGA14-*lgt*. As a negative control we complemented the Δ*lgt* mutant with vector lacking the *lgt* insert, generating Δ*lgt*::pGA14-*cm*. Inactivation of *lgt* resulted in viable *S. suis* bacteria able to grow efficiently in THB after a slightly increased lag phase (Fig. 3).

**Figure 4:**
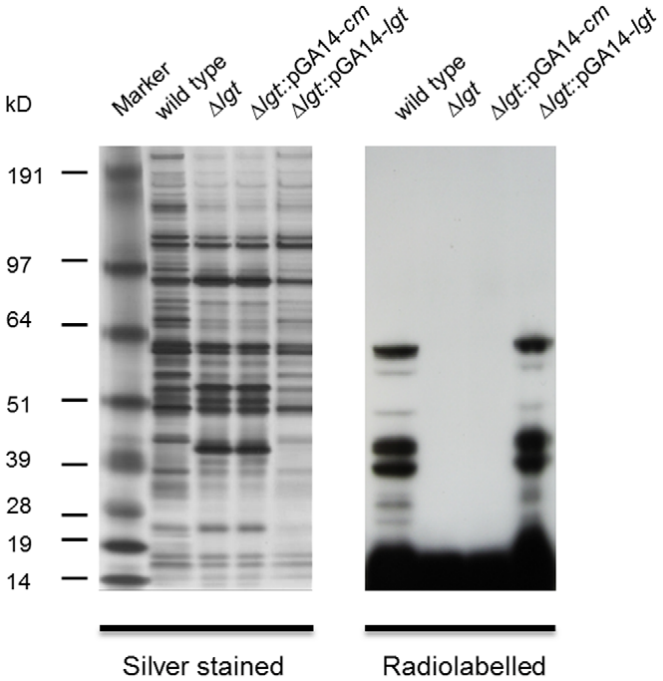
Lipidation of wild type and Δ*lgt* mutant bacteria. Lipidation was assessed by incubating the wild type and mutant bacteria with [^3^H]palmitic acid, followed by penicillin treatment and SDS-PAGE. Lipidation was visualized using autoradiography. As a control, total protein release of wild type and mutant bacteria was visualized with Silver staining.

To verify that lipoprotein processing had been abolished in the Δ*lgt* mutant, lipidation of lipoproteins in the wild type and mutant was analyzed. Bacteria were grown in the presence of [^3^H]palmitic acid and subsequently treated with penicillin. Similar amounts of protein were released from wild type and (complemented) Δ*lgt* mutant bacteria (Fig. 4). Several radiolabeled proteins were detected in the supernatant of the wild type and the Δ*lgt*::pGA14-*lgt* mutant (Fig. 4), whereas no radiolabeled (lipo)proteins were detected in the supernatant of the Δ*lgt* mutant and the Δ*lgt*::pGA14-*cm* mutant. These data confirm that Lgt is responsible for lipid modification of prelipoproteins in *S. suis*.

### Disruption of *lgt* abolishes activation of human TLR2/6

**Figure 5:**
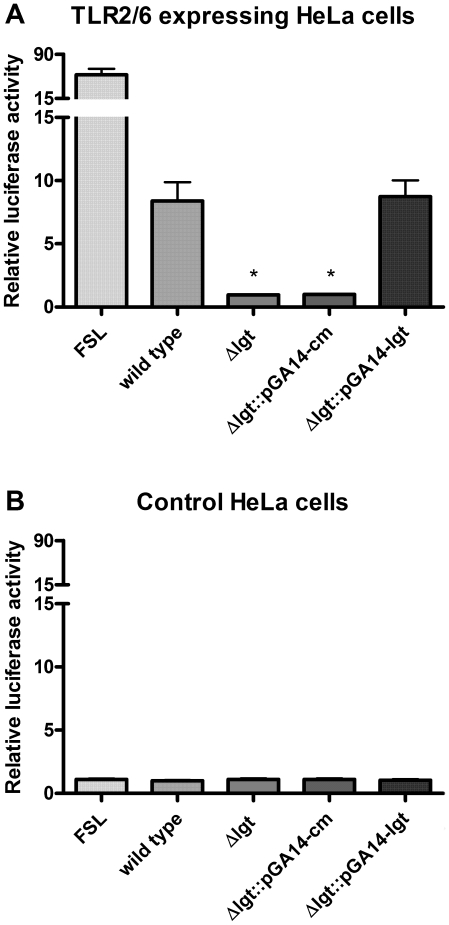
TLR2/6 activating capacity of wild type and Δ*lgt* mutant bacteria. HeLa 57A cells expressing human TLR2/6 (A) and control cells transfected with vector without insert (B) were stimulated with wild type, Δ*lgt*, Δ*lgt*::pGA14-*cm*, and Δ*lgt*::pGA14-*lgt* mutant bacteria in the presence of penicillin (30 µg/ml). At 5 h post stimulation, NF-κB luciferase activity was determined. The diacylated lipopeptide FSL was used as a positive control. Data represent relative luciferase activity calculated by dividing the normalized activity of the test samples by the normalized activity of medium-stimulated negative control samples. Values represent the mean ± SD of three independent experiments performed in duplicate. * P<0.05 compared to wild type level.

To investigate whether lipid modification of *S. suis* prelipoproteins is a prerequisite for human TLR2/6 activation, we compared the abilities of the (penicillin-treated) wild type, Δ*lgt* mutant and the complemented Δ*lgt* mutant strains to activate HeLa cells expressing human TLR2/6. Both *S. suis* wild type and the Δ*lgt*::pGA14-*lgt* mutant induced significant TLR2/6 activation (Fig. 5A), in contrast to the Δ*lgt* mutant and the Δ*lgt*::pGA14-*cm* strain. In all cases, stimulation of HeLa cells transfected with the vectors lacking the TLR gene yielded only background levels of NF-κB activity (Fig. 5B). These data indicate that the presence of a protein bound lipid moiety is a prerequisite for activation of human TLR2/6 and that the *S. suis* lipoproteins are the primary ligands that activate the human TLR2/6 complex.

### Inactivation of *lgt* reduces PBMC activation

**Figure 6:**
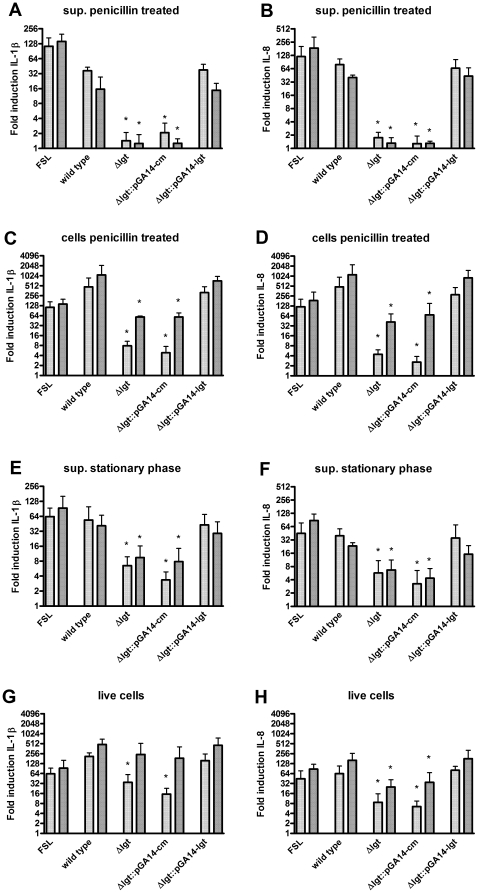
Porcine PBMC activating capacity of wild type and Δ*lgt* mutant bacteria. Porcine PBMCs were stimulated with wild type, Δ*lgt*, Δ*lgt*::pGA14-*cm*, and Δ*lgt*::pGA14-*lgt* mutant bacteria. PBMCs were stimulated with supernatants derived from penicillin treated bacteria (A, B), cells in the presence of penicillin (C, D), supernatants of stationary phase grown bacteria in the absence of penicillin (E, F) and cells in the absence of penicillin (G, H). At 2 h (light grey bar) and 4 h (dark grey bar) after stimulation, IL-1β (A, C, E, G) and IL-8 (B, D, F, H) mRNA levels were determined. The diacylated lipopeptide FSL was used as a positive control. Data represent relative fold activation calculated by dividing the normalized activity of the test samples by the normalized activity of medium-stimulated negative control samples. Values represent the mean ± SD of three independent experiments performed in duplicate. * P<0.05 compared to wild type level.

In contrast to the transfected HeLa cells expressing human TLR2/6, porcine PBMC express multiple innate immune receptors that may respond to various *S. suis* components. To assess the contribution of lipoproteins to PBMC activation, we stimulated porcine PBMCs with (penicillin-treated) supernatants and cells of wild type, Δ*lgt* mutant, Δ*lgt*::pGA14-*cm* mutant and Δ*lgt*::pGA14-*lgt* mutant bacteria. Stimulation with the wild type and the Δ*lgt*::pGA14-*lgt* mutant bacterial supernatants resulted in efficient induction of IL-1β and IL-8 mRNA at 2 h and 4 h post stimulation (Figs. 6A and B). As expected, only minimal induction of IL-1β and IL-8 mRNA was observed after stimulation with the Δ*lgt* mutant and Δ*lgt*::pGA14-*cm* mutant derived supernatant. In line with the activation kinetics of the supernatants, PBMCs stimulation with wild type and Δ*lgt*::pGA14-*lgt* mutant bacteria also resulted in efficient induction of IL-1β and IL-8 mRNA at 2 h and 4 h post stimulation (Fig. 6C and D). The IL-1β and IL-8 mRNA levels induced by the Δ*lgt* mutant and the Δ*lgt*::pGA14-*cm* mutant were once more strongly reduced compared to the wild type strain especially at 2 h post stimulation. These results suggest *S. suis* lipoproteins as the principal activators of the porcine PBMC innate immune response.

### Contribution lipoproteins in activating porcine PBMCs in the absence of penicillin

The above results were obtained with penicillin-treated *S. suis* to enhance the release, and enable the identification, of immune activating bacterial factors. To assess the contribution of lipoproteins as activators of the porcine PBMC response in the absence of antibiotics, we stimulated porcine PBMCs with live *S. suis* and supernatants of *S. suis* grown to stationary phase without penicillin. Stimulation of PBMCs with both cells and supernatant of wild type and Δ*lgt*::pGA14-*lgt* mutant bacteria resulted in efficient induction of IL-1β and IL-8 mRNA (Fig. 6E–H). Much less induction of IL-1β and IL-8 mRNA was observed after stimulation with the Δ*lgt* mutant and the Δ*lgt*::pGA14-*cm* mutant cells and supernatants, consistent with the results obtained in the presence of penicillin. Together, these results indicate that *S. suis* lipoproteins are major activators of the innate immune system of the pig.

## Discussion

In the present study we identified 9 *S. suis* lipoproteins within a fraction able to activate porcine PBMCs efficiently. Disruption of the *lgt* gene required for lipoprotein synthesis strongly reduced activation of porcine PBMCs. This effect was restored after complementation of the gene defect. Altogether, these results provide conclusive evidence that lipoproteins are potent and dominant innate immunity activating molecules of *S. suis*.

The identification of *S. suis* lipoproteins as major activators of porcine PBMCs resulted from detailed analysis of active fractions of bacterial culture supernatant. Mass spectrometry results and the finding that similar fractions activated porcine PBMCs and the human TLR2/6 complex pointed towards possible lipoproteins as activating molecules. We possibly only identified the most abundantly expressed or released lipoproteins of *S. suis* by mass spectrometry. As shown for several bacterial species including *S. suis*, expression levels may vary between different lipoproteins and are influenced by the bacterial environment. In a recent study, three divalent-cation-binding lipoproteins of *S. suis* were shown to be up regulated after divalent-cation deprivation *in vitro* [24] and a fourth divalent-cation-binding lipoprotein was shown to be up regulated in mice [20]. Of the 9 lipoproteins we identified here, the basic membrane lipoprotein (SSU0934 in *S. suis* P1/7) and a putative high affinity metal binding lipoprotein (SSU1869 in *S. suis* P1/7) have been demonstrated to be recognized by convalescent pig sera [19], [20], indicating their expression and immunogenicity *in vivo*.

Porcine PBMCs are expected to express a wide range of PRRs including TLRs. Activation of TLRs by bacterial PAMPs generally results in nuclear translocation of NF-κB followed by transcription of pro-inflammatory cytokines and chemokines such as IL-1β and IL-8. Efficient activation and differences in transcript levels of IL-1β and IL-8 mRNA were already observed at a *S. suis* to PBMC ratio of 1∶1 at the start of infection and as early as 2 and 4 h post infection. As expected, other NF-κB dependent cytokines, such as IL-6, TNF-α and IL-10 showed similar kinetics when compared to IL-1β and IL-8 expression profiles (Fig. S1). Cell damage at prolonged infection prevented measurements of accurate cytokine release into the medium. The increase in IL-1β and IL-8 in the porcine PBMCs is likely mediated via porcine TLR2/6, although this could not be measured as the porcine TLR read out systems have not been validated in a porcine cell background. However, we successfully demonstrated that the identified *S. suis* lipoproteins activate human TLR2/6, which has a high level of sequence identity with porcine TLR2/6.

During this study, we initially used penicillin to enhance the release of possible innate immunity activating components of *S. suis*. Penicillin inactivates the penicillin binding proteins essential for the crosslinking of bacterial peptidoglycan, skewing the release of components normally tightly attached to the cell wall or cell membrane. This procedure resulted in increased release of (lipo)proteins in the culture supernatant and facilitated the identification of the innate immunity activating (lipo)proteins. The effect of increased innate sensing after penicillin treatment has also been reported for *S. pneumoniae* [25]. Our finding that PBMC activation also occurred in the absence of penicillin (Fig. 6) excludes adverse effects of penicillin (e.g. cell lysis) on the immune activation. In the absence of penicillin, the effects of immune activation were most pronounced during stationary growth phase. This likely explains the lack of activation of human TLR2/6 by logarithmic phase-derived bacteria [12].

In *S. suis* a significant group of lipoproteins is predicted to have substrate binding and transport functions (Table S1) which suggests lipoproteins to be involved in nutrient acquisition. Interestingly, the *in vitro* growth data of the Δ*lgt* mutant bacteria suggest that nutrient acquisition mediated by lipoproteins is not critical for growth in rich media such as THB or that lipid modification of lipoproteins is not essential for lipoprotein function. The *in vitro* growth ability of the *S. suis* Δ*lgt* mutant bacteria resembles observation of several other *lgt* mutants in Streptococcal species such as *S. pneumoniae*, *S. equi*, *S. agalactiae*, *S. sanguinis* and *S. uberis* [17], [26], [27], [28], [29]. Probably lipoproteins without lipid moiety are still anchored in the bacterial membrane and able to fulfill (partly) their roles in nutrient acquisition. The observations of reduced innate immune activation, observed for the *S. suis* Δ*lgt* mutant strain, is in agreement with observations in other Gram-positive bacterial species, including *Staphyloccoccus aureus*, *Listeria monocytogenes* and *S. agalactiae* [17], [18], [30].

On the basis of our results, the absence of lipoprotein lipidation may benefit *S. suis* as it may aid to evade sensing by the innate immune system. On the other hand, the absence of lipoprotein lipidation might affect lipoprotein functionality, which may affect *in vivo* growth and virulence characteristics, interactions with components in the host, and interactions with other surrounding *S. suis* bacteria. These growth effects complicate the interpretation of *in vivo* studies on the effect of *S. suis* on the innate immune response. In *S. sanguinis* and *S. pneumoniae* inactivation of Lgt processing of lipoproteins have been shown to moderately reduce virulence [26], [27]. In *S. agalactiae* and *S. aureus* Δ*lgt* mutant bacteria became hypervirulent at a low dose [17], [31]. Whether virulence of the *S. suis* Δ*lgt* mutant is affected compared to wild type bacteria and whether this is caused by an altered innate immune response or growth characteristics awaits further study.

## Materials and Methods

### Ethics Statement

Fresh porcine blood was obtained in accordance with a protocol (2008120.a) approved by the Animal Experiments Committee of the Central Veterinary Institute (Lelystad, The Netherlands), in agreement with the Dutch Experiments on Animals Act (Project code: 2008149).

### Bacterial strains and growth conditions

In this study we used a serotype 9 strain (strain 8067, virulent pig isolate, Smith *et al.*, unpublished results), which is previously shown to activate the innate immune system via human TLR2/6 more efficiently compared to serotype 2 strains [12]. Wild type bacteria, isogenic mutants as well as complemented mutant strains were grown on Colombia agar plates (Oxoid Ltd, London, United Kingdom) containing 6% horse blood at 5% CO_2_ and 37°C. Liquid cultures were grown in Todd-Hewitt broth (THB) (Oxoid Ltd.) for 18 h at 37°C without agitation. *Escherichia coli* were grown on Luria-Bertani (LB) agar plates or in LB broth. When necessary, antibiotics were added to culture media at the following concentrations: for *E. coli*, ampicillin 100 µg/ml; chloramphenicol 8 µg/ml and spectinomycin 100 µg/ml; for *S. suis*, chloramphenicol 5 µg/ml and spectinomycin 100 µg/ml. For use in stimulation experiments, bacteria were pelleted by centrifugation at 4,500× *g* for 10 min and resuspended to 1.0×10^9^ CFU/ml in Dulbecco's phosphate buffered saline (D-PBS).

### General DNA techniques

Genomic DNA from *S. suis* was isolated as described previously [32]. PCRs were conducted with Phusion High-Fidelity DNA polymerase (BIOKE, Leiden, The Netherlands). Plasmid DNA was isolated with the Plasmid DNA Purification System (Promega, Leiden, The Netherlands). DNA purifications were performed with the Zymogen clean up kits (BaseClear, Leiden, The Netherlands). Ligations were performed with T4 DNA ligase (Promega) and ligation mixtures were used to transform *E. coli*. Plasmids were introduced into *S. suis* via electroporation [33].

### Generation of Δ*lgt* mutant

Primers used in this study are listed in Table S2. Primers 1 and 4 were used to amplify a fragment of the chromosomal DNA of strain 8067 containing the intact *lgt* gene flanked on both sides by 1.5 kb regions. This fragment was ligated to the blunt cloning vector pJET1.2 (Fermentas, St. Leon-Rot, Germany) and ligation mixtures were transformed to *E. coli*. Plasmid DNA (designated pJET-*lgt*) obtained from transformants was then used to replace an internal fragment (about 300 bp) of *lgt* by a Spc resistance cassette. To do this, we used an inverse PCR strategy on pJET-*lgt* using primers 2 and 3. In addition, the Spc cassette was amplified from pGA14-*spc* [22] using primers 9 and 10. The amplified fragments were digested with *Xma*I and *Sal*I and ligated together. Ligation mixtures were introduced into *E. coli* to generate pJET-*lgt*-*spc*. The entire insert fragment of pJET-*lgt*-*spc* was subsequently amplified using primers 1 and 4 and ligated to the thermo sensitive shuttle vector pSET5 [34], which was linerialized with the *Sma*I restriction enzyme, generating pSET5-*lgt*-*spc*. The pSET5-*lgt*-*spc* plasmid was then introduced into *S. suis* strain 8067 by electroporation and transformants were selected on Columbia agar plates at 30°C in the presence of spectinomycin. Several individual colonies were grown overnight in THB (10 ml) containing spectinomycin at 30°C. The overnight cultures were then diluted 1∶100 in THB without antibiotics and incubated for 4 h at 38°C. Cultures were serially diluted on Columbia agar plates containing spectinomycin at 38°C to select for chromosomal integration. Individual colonies that had lost the vector mediated chloramphenicol resistance were confirmed to have the expected mutant genotype by PCR using primer pairs 5,6 and 7,8 as well as by Southern blotting.

### Complementation of the Δ*lgt* mutant

To complement the Δ*lgt* mutant with an intact *lgt* gene, we constructed an expression plasmid containing the wild type *lgt* gene including its putative promoter. Primers 13 and 14 were used to amplify the *lgt* fragment, which was cloned into pJET1.2 generating pJET1.2-*lgt*-expr. Subsequently, pJET1.2-*lgt*-expr was digested with *Sma*I and *Sal*I and the *lgt* fragment was purified and cloned into pGA14 [35] digested with *Sma*I and *Sal*I, generating pGA14-*lgt*-expr. Finally, the chloramphenicol resistance gene (*cm*) of pSET5, amplified with primers 15 and 16 and digested with *Sal*I, was introduced at the *Sal*I site of pGA14-*lgt*-expr to yield pGA14-*lgt*-expr-*cm*. As a negative control, *cm* was introduced in pGA14 digested with *Sal*I, generating pGA14-*cm*. Both plasmids were subsequently introduced into the Δ*lgt* mutant generating Δ*lgt*::pGA14-*lgt* and Δ*lgt*::pGA14-*cm* respectively. RNA expression of the *lgt* gene in the Δ*lgt*::pGA14-*lgt* mutant was confirmed by quantitative real time PCR.

### Growth analysis

Overnight cultures of wild type and mutant bacteria were 1∶100 diluted in fresh THB and optical density at 600 nm (OD_600_) of 400 µl samples was followed in time using the Bioscreen C (Thermo Scientific, Breda, The Netherlands) at 37°C. Overnight cultures of wild type, Δ*lgt* mutant and complemented mutants had similar OD_600_ values and contained the same amounts of CFU.

### [^3^H]palmitate labeling

Bacteria were grown for 18 h at 37°C in THB, pelleted, resuspended to 1.0×10^9^ CFU/ml in D-PBS and then diluted 1∶20 in chemical defined medium (CDM) consisting of a 1∶1 mixture of HAM-F12 nutrient mixture (Invitrogen, Breda, The Netherlands) and NCTC-109 medium (Sigma-Aldrich, Zwijndrecht, The Netherlands) containing 10 µCi/ml [9,10-3^H^]palmitic acid (Perkin Elmer, Groningen, The Netherlands). At an optical density of 0.4 (600 nm), penicillin G (Sigma-Aldrich) was added to the culture to a final concentration of 30 µg/ml. After 2 h of incubation at 37°C, the bacteria and medium were separated by centrifugation (4,500×*g*, 10 min) and the supernatant was 40 times volume concentrated by Amicon Ultra-15 centrifugal filter devices with a 3 kD cut-off (Millipore, Amsterdam, The Netherlands). Subsequently, LDS Sample Buffer (Invitrogen) was added to the concentrated fraction and 30 µl samples were separated using SDS-PAGE. Finally, the gel was fixed, dried, and exposed to an autoradiography film for 24 h.

### Generation of (concentrated) bacterial supernatant

Wild type 8067, Δ*lgt* mutant and complemented Δ*lgt* mutant strains were grown for 18 h at 37°C in THB, pelleted, resuspended to 1.0×10^9^ CFU/ml in D-PBS and diluted 1∶20 in CDM. Penicillin treated supernatant was obtained by adding penicillin G (final concentration of 30 µg/ml) to cultures when OD_600_ values reached 0.4. After 2 h of incubation at 37°C, the bacteria and medium were separated by centrifugation (4,500×*g*, 10 min) and the supernatants were 0.2 µm filter sterilized. Stationary phase-derived supernatant was obtained by incubating 1∶20 diluted CDM cultures for 24 h at 37°C followed by centrifugation (4,500×*g*, 10 min) and filtration (0.2 µm). Supernatants were directly used for PBMC stimulation or used for further concentration. For this, 10% TCA w/v was added to the supernatants followed by overnight incubation at 4°C. After centrifugation at 30,000× *g* for 30 min, the pellets were washed with 100% acetone and air dried. Finally, protein pellets were dissolved in LDS Sample Buffer.

### Identification of proteins within crude immune stimulatory fraction

Proteins (1 mg) present in a concentrated bacterial supernatant of *S. suis* strain 8067 were separated on a 10% SDS-polyacrylamide gel (15 cm in length, 12 cm wide) under non-reducing conditions (no boiling). One cm of the gel was stained with the Silver staining kit Plus One from GE Healthcare (Uppsala, Sweden) and the remaining gel was cut into 0.5 cm strips of 24 different molecular size ranges. Each strip was homogenized with a mortar in a 1% w/v SDS solution to solubilize proteins. Gel residue was removed by centrifugation (12,000×*g*, 10 min) and five volumes of cold acetone were added to the supernatant. After overnight incubation at −20°C, precipitate was collected by centrifugation (12,000×*g* 4°C for 10 min). Pellets were dissolved in 200 µl of 10 mM Tris-HCl pH 8. Fractions (50 µl) were tested for activity using porcine PBMCs and human TLR2/6 expressing HeLa 57A cells. Fractions that showed >5 fold porcine PBMC activation were once more separated on a 4–12% polyacrylamide gel (Invitrogen), stained with SimpleBlue Safe Stain (Invitrogen), excised from the gel, and identified with mass spectrometry. Briefly, proteins were reduced with dithiothreitol, alkylated with iodoacetamide, and digested with trypsin (Roche) as described [36]. Samples were subjected to nanoflow LC (Eksigent) using C18 reverse phase trap columns (Phenomenex; column dimensions 2 cm×100 µm, packed in-house) and subsequently separated on C18 analytical columns (Reprosil; column dimensions, 20 cm×50 µm; packed in-house) using a linear gradient from 0 to 40% B (A = 0.1 M acetic acid; B = 95% (v/v) acetonitrile, 0.1 M acetic acid) in 60 min and at a constant flow rate of 150 nl/min. Column eluate was directly coupled to a LTQ-Orbitrap-XL mass spectrometer (Thermo Fisher Scientific) operating in positive mode, using Lock spray internal calibration. Data were processed and subjected to database searches using MASCOT software (Matrixscience) against Swiss Prot and non-redundant NCBI database with a 10 ppm mass tolerance of precursor and 0.8 Da for the fragment ion.

### PBMC isolation and stimulation

Blood of three to four week old piglets from a specific pathogen free (SPF) herd was aseptically collected and mixed with heparin (LEO Pharma, Breda, Netherlands) to a final concentration of 5 IE/ml. Subsequently, PBMCs were isolated with lymphoprep tubes (Lucron Bioproducts, Gennep, Netherlands), according the manufactures instructions. The PBMCs were resuspended to 5.0×10^6^ cells/ml in RPMI 1640 supplemented with 2% v/v of homologous serum (from the same animal as the PBMCs) and 30 µg/ml of penicillin. Cells (1 ml) were seeded into 24 well tissue plates. After overnight incubation, cells were stimulated with 50 µl of SDS-PAGE derived fractions, 5.0×10^6^ *S. suis* bacteria (in presence or absence penicillin), or 50 µl of *S. suis* derived bacterial supernatant. After stimulation (2 and 4 h) cells were lysed and frozen (−80°C) and stored until RNA isolation and cytokine detection. We used quantitative real time PCR analysis, because the available porcine cytokine ELISAs are much less sensitive, particularly for stimulation experiments that last only 2–4 h. FSL-1 (100 ng/ml) and medium-stimulated cells served as positive and negative controls, respectively.

### RNA isolation, cDNA synthesis, and quantitative real time PCR

Total RNA was isolated with the High Pure RNA Isolation Kit (Roche Diagnostics, Mannheim, Germany), according the manufactures instructions. RNA quantity and quality was checked with the NANOdrop (Thermo Fisher Scientific, Pitsburgh, USA). To make cDNA, 200 ng RNA was reverse transcribed using OligoDt and Superscript III (Promega), according the manufactures instructions. For quantitative real time PCR analysis of IL-1β and IL-8 cytokines, 5 µl of 20 times diluted cDNA was added to 1×power cyber green mixture (Applied Biosystems, Nieuwe Kerk aan de IJssel, The Netherlands) containing 0.625 µM of forward and reverse primer (Table S2) in a total of 20 µl. Serial dilutions of pGemTeasy plasmids containing the PCR fragment of interest were used as internal standards. The PCR was performed on a 7500 Fast Real-Time PCR system (Applied Biosystems). The PCR program consisted of a denaturation step at 95°C for 10 min followed by 40 cycles of denaturation at 95°C for 15 sec, annealing at 59°C for 30 sec, and elongation at 72°C for 36 sec. Ct values for the tested cytokines in each sample were expressed as cDNA quantity (ng) using the internal standards. Subsequently, the IL-1β and IL-8 ng levels were normalized with the ng levels of the house keeping gene *gapdh*. To calculate fold inductions, normalized IL-1β and IL-8 levels of stimulated cells were divided by normalized IL-1β and IL-8 levels of medium-stimulated control cells.

### Stimulation of human TLR2/6 transfected HeLa cells

The HeLa 57A cell line, stably transfected with a NF-κB luciferase reporter construct [37], was generously provided by Dr. R.T. Hay (Institute of Biomolecular Sciences, University of St. Andrews, St. Andrews, Scotland, UK). Cells were propagated in Dulbecco's modified Eagle's medium (DMEM) supplemented with 10% fetal bovine serum (FBS) and incubated at 37°C and 10% CO_2_. For transfection experiments, cells were seeded in 48 well tissue culture plates. When 50% confluence was reached, cells were transfected with 250 ng DNA/well using FuGENE 6 (Roche Diagnostics, Almere, The Netherlands) at a lipid to DNA ratio of 3 to 1. For TLR2/6 transfection, expression plasmids carrying the human TLR2, human TLR6 and human CD14 gene [22] were used, kindly provided by Dr. A.M. Keestra (Utrecht University, The Netherlands). Cells transfected with empty vector were used as negative controls and the pTK-LacZ vector was used for normalization of the transfection efficiency. After 48 h of incubation at 37°C, medium was replaced with fresh medium containing 30 µg/ml penicillin (Sigma-Aldrich). Subsequently, cells were stimulated for 5 h with 2.0×10^7^/ml bacteria or with 50 µl of SDS-PAGE derived cell wall fractions. The di-acylated lipopeptide FSL-1 (InvivoGen, Toulouse, France) (100 ng/ml) served as a TLR2/6 specific control. After stimulation, cells were washed twice with D-PBS and lysed in 0.1 ml of passive Reporter Lysis Buffer (Promega), according the manufactures description. Subsequently, luciferase activity was determined with a Victor 1420 multilabel counter (PerkinElmer, Groningen, The Netherlands) by incubating 20 µl of lysed cells with 50 µl of luciferase assay substrate (Promega). Luciferase activity was normalized for transfection efficiency by determination of ß-galactosidase activity with the ß-galactosidase assay (Promega). Relative fold activation was calculated as the normalized reporter activity of the test samples divided by the normalized activity of medium-stimulated control cells.

### Statistical analysis

Statistical analysis was performed in GraphPad Prism. Normal distribution of data was evaluated using Kolmogorov-Smirnov test. Subsequently, normal distributed data were analyzed using an unpaired Students's *t* test and non-normal distributed data were analyzed using the Mann-Whitney test. P-values <0.05 were taken as significant.

## 

## Supporting Information

Figure S1 **IL-6, TNF-α and IL-10 cytokine responses of porcine PBMCs stimulated with wild type** ***S. suis*** **.** Porcine PBMCs were stimulated with whole *S. suis* strain 8067 bacteria (cells) in the presence of penicillin or with supernatant (sup.) derived from penicillin treated bacteria. At 2 (light grey bar) and 4 h (dark grey bar) post stimulation IL-6 (A), TNF-α (B) and IL-10 (C) mRNA expression levels were determined by quantitative real time PCR. The diacylated lipopeptide FSL was used as a positive control. Data represent fold inductions calculated by dividing the normalized cytokine levels of stimulated cells by the normalized cytokine levels of medium-stimulated negative control cells. Values represent the mean ± SD of two experiments performed in duplicate.
(TIF)

Table S1 **Putative lipoproteins of** ***S. suis*** **strain P1/7.**
(DOC)

Table S2 **Primer sequences.**
(DOC)
