# Supplementary material for: Lgt Processing Is an Essential Step in Streptococcus suis Lipoprotein Mediated Innate Immune Activation
Source: PLoS One. 2011 Jul 19;6(7):e22299. doi: 10.1371/journal.pone.0022299 (PMC3139625; doi:10.1371/journal.pone.0022299)
Supplement: Table S1 — Putative lipoproteins of S. suis strain P1/7. (DOC) [file pone.0022299.s002.doc]

**Table S1.** Putative lipoproteins of *S. suis* strain P1/7

**Bold: Identified in porcine PBMC stimulating fraction.**
