# Supplementary material for: Lgt Processing Is an Essential Step in Streptococcus suis Lipoprotein Mediated Innate Immune Activation
Source: PLoS One. 2011 Jul 19;6(7):e22299. doi: 10.1371/journal.pone.0022299 (PMC3139625; doi:10.1371/journal.pone.0022299)
Supplement: Table S2 — Primer sequences. (DOC) [file pone.0022299.s003.doc]

**Table S2.** Primer sequences

Sequences in *Italic* correspond to restriction sites
